# Factors of the Ecosystem Service Value in Water Conservation Areas Considering the Natural Environment and Human Activities: A Case Study of Funiu Mountain, China

**DOI:** 10.3390/ijerph182111074

**Published:** 2021-10-21

**Authors:** Chunyang Guo, Jianhua Gao, Boyan Zhou, Jie Yang

**Affiliations:** 1 College of Geography and Environmental Science, Henan University, Kaifeng 475004, China; 104752170025@vip.henu.edu.cn (C.G.); 104752180028@vip.henu.edu.cn (B.Z.); yangjie@henu.edu.cn (J.Y.); 2 Key Laboratory of Geospatial Technology for the Middle and Lower Yellow River Regions, College of Geography and Environmental Science, Henan University, Kaifeng 475004, China; 3 Key Research Institute of Yellow River Civilization and Sustainable Development & Collaborative Innovation Center on Yellow River Civilization Jointly Built by Henan Province and Ministry of Education, Henan University, Kaifeng 475001, China

**Keywords:** ecosystem services, random forest, influencing factors, water conservation area

## Abstract

Water conservation areas play an important role in regional ecological security patterns. The Funiu Mountain water conservation area is located in the densely populated central region of China, where human disturbance to the ecosystem is strong and ecosystem services are facing a very serious situation. Identifying and evaluating the factors leading to changes in the ecosystem service value (ESV) of the Funiu Mountain water conservation area can provide scientific guidance for ecological management and sustainable development. Using multi-source data and machine learning methods, our research reveals the characteristics of the spatio-temporal variation in the ESV, constructs a system of ESV influencing factors from the comprehensive perspectives of the natural environment and human activities, and discusses the comprehensive effects of the influencing factors on the Funiu Mountain area from 2000 to 2015. The results are as follows. (1) From 2000 to 2005, the ESV increased 375 million yuan, and from 2005 to 2015, it decreased 154 million yuan. (2) Hydrological regulation, biodiversity maintenance, soil conservation, gas regulation, and climate regulation were the main types of ecosystem services in the Funiu Mountain area. (3) The ESV was influenced by the comprehensive effects of the natural environment and human activities. Population was the most important influencing factor of the ESV; in addition, the normalized difference vegetation index (NDVI), precipitation, and economic factors had important influences on the ESV. (4) With the intensification of human activities, humanistic factors have surpassed the relatively stable natural factors, becoming the main factors of the ESV. With economic development, the effect of human activities on the ESV may be further intensified in the future.

## 1. Introduction

The ecosystem is the foundation of human survival and development. While the ecosystem provides humans with fresh water, food, medicinal products, and other means of industrial and agricultural production, it also maintains a balance between atmospheric, hydrological, and biogeochemical factors [1]. In recent centuries, with the process of industrialization, human intervention in nature has increased [2]. Deforestation, wetland development, the exploitation and utilization of biological resources, and changes in land use patterns have led to great changes in global ecosystem patterns [3]. The area of the natural ecosystem has decreased, and the area of the human-controlled ecosystem has rapidly increased. At the same time, a large number of environmental pollutants have entered the ecosystem, greatly exceeding the carrying capacity of the ecosystem and destroying the structure and functions of the ecosystem, resulting in damage to ecosystem service functions [4]. The ability of the ecosystem to regulate the atmospheric chemical environment, preserve biodiversity and the evolutionary process, and maintain soil fertility has been weakened, leading to the global ecological crisis and threatening the future development of human beings [5]. In its “Millennium Ecosystem Assessment” published in 2005 [6], the United Nations noted that of the 24 identified ecosystem services, 15 (approximately 60%) were in a state of continuous degradation. In the next 50 years, if no action is taken there will be no chance to reverse those changes and even more of the ecosystem will be destroyed [7].

The ecosystem service value (ESV) is an important indicator used to measure regional sustainable development. Clarifying the influencing factors of ecosystem service functions is a prerequisite for rational ESV management, the regulation of ecosystem service functions, and optimal decision making [8,9]. Thus, this topic has attracted the attention of many ecologists and economists.

ESV assessment mainly quantifies the value of the ecological capital, biodiversity, and natural capital in the ecosystem [10] using a variety of methods. These methods can be roughly divided into two categories: the functional value method [11,12,13,14] and the equivalent factor method [15,16]. The functional value method constructs a series of ecological equations and adjusts a large number of ecological parameters. The calculation process is more complicated and suitable for small-scale research [17,18]. The equivalent factor method can use an alternative market method or simulated market method to calculate the values of different ecosystem service functions [19,20]. This method is intuitive, simple, and suitable for regional and global scale assessments, and therefore, it is widely used in research [21,22,23,24,25].

Ecosystem services serve as a bridge connecting the natural environment and human well-being. Analyzing the influencing factors of ESV helps to objectively understand the formation mechanism of ecosystem services. Such analysis is an important part of carrying out the scientific management of regional ecosystems, coordinating the relationship between humans and land, and realizing sustainable social and economic development [26,27]. In this regard, scholars have conducted much research. Some studies state that socioeconomic factors are the key to ESV changes and discuss direct driving factors such as land use change [28,29] and indirect driving factors such as population [30], the economy [31], urbanization [32,33], and policy [34,35]. Some studies also believe that natural factors such as terrain [36,37] and climate [38] are the main reasons for ESV changes. We hold that ecosystem services are a complex process driven by multiple factors. These factors involve nature, the economy, society, and many other aspects [39,40]. It is inadequate to analyze the impact of human activities on ecosystem services alone or to analyze the impact of natural environment on ecosystem services alone. Therefore, comprehensively considering the impact of the natural environment and human activities on the ESV should be a new research direction.

In China, the Funiu Mountain area is the headwaters of some tributaries of the Yangtze River, Yellow River, and Huaihe River. At the same time, the Danjiangkou Reservoir in the Funiu Mountain area is the water source of the middle route of China’s South-to-North Water Diversion Project. As an important water conservation area, Funiu Mountain bears very important ecological functions. Different from other water conservation areas, the Funiu Mountain water conservation area is located in the central region of China, where the population is relatively dense. Human activities strongly disturb the ecosystem, weakening the structure and function of the original ecosystem. An analysis that considers natural factors in isolation may not reflect the actual influencing mechanism of the real ecosystem in the Funiu Mountain water conservation area. Therefore, it is important to explore the combined effects of the natural environment and human activities to gain an in-depth and accurate understanding of the mechanism of ecosystem service functions.

Natural data are mostly based on geographical units, and human-related data are mostly based on administrative units. The spatial dislocation of data will inevitably affect related quantitative research and become a bottleneck for research on the influencing factors of the ESV. Emerging research focuses on the spatial refinement (grid) of socioeconomic data, going beyond the barriers of data fusion. This approach makes possible the spatial matching and organic integration of natural data and socioeconomic data, greatly improving the accuracy of the research results. Notably, integrating natural data and socioeconomic data on a spatial grid scale will inevitably generate large amounts of data, which will far exceed the data processing capacity of traditional analytical models of influencing factors. The recent rapid development of machine learning methods provides effective technical support for such research. Among them, the random forest (RF) algorithm is a very important method in machine learning and can be used for data mining and classification regression. Existing research shows that the RF algorithm can handle high-dimensional discrete or continuous data and has a high tolerance for missing data. In addition, due to the introduction of sample randomness and feature randomness, it has a certain anti-noise ability, and it does not easily fall into overfitting during operation. More importantly, RF algorithm is not affected by the multicollinearity problem encountered in general regression analysis. In addition, there is no need to make variable selection. The algorithm represents a convenient method of calculating the nonlinear effects of variables and can evaluate the importance of independent variables [41,42]. Thus, this method has good applicability for regression analysis based on big data.

In response to the research background above, we attempt to improve and explore the following aspects. The specific contributions of this study are as follows: (1) Based on a grid scale of 1 km × 1 km, we calculated the ESV of the Funiu Mountain water conservation area from 2000 to 2015 by using the equivalent factor method and analysed the characteristics of the area’s spatio-temporal evolution. (2) On the basis of exploring the influencing mechanism of the ESV, we constructed a framework of the influencing factors of the ESV from the comprehensive perspective of the natural environment and human activities. (3) We used the RF algorithm to clarify the dominant factors of ESV changes. Our attempt is to provide a new perspective to understand the influencing mechanism of the ESV, and its results can provide a decision-making basis for ecological civilization construction in China.

## 2. Materials and Methods

### 2.1. Study Area

**Figure 1:**
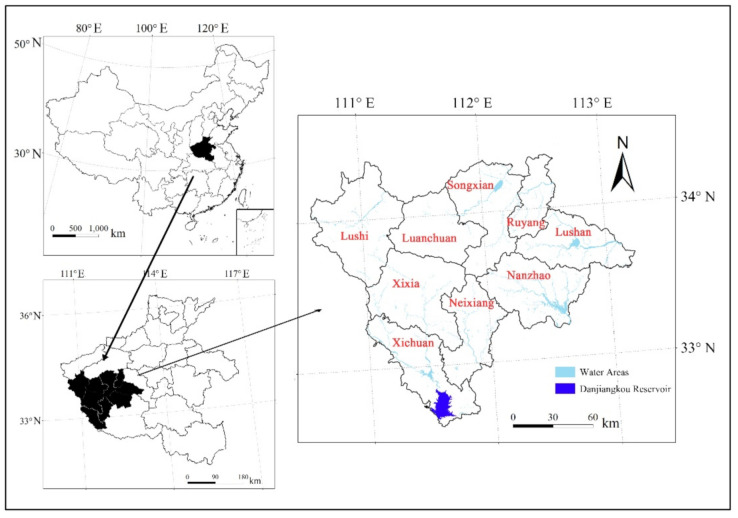
Location of the Funiu Mountain water conservation area.

The Funiu Mountain water conservation area is located in the southwest of Henan Province, China (110°30′–113°30′ E, 32°10′–34°15′ N). It is the headwaters of some tributaries of the Yangtze River, Yellow River, and Huaihe River. Additionally, it is the water source of the middle route of China’s South-to-North Water Diversion Project and plays an important role in water conservation (Figure 1). The landform types in the Funiu Mountain water conservation area are plains, hills, and mountains, and the main soil types are yellow-brown, brown, and yellow soil [43]. The climate is transitional from north subtropical to warm temperate, and the average annual temperature is 13.6–15.1 °C. The study area encompasses nine counties: including Lushi County in Sanmenxia city; Luanchuan County, Songxian County and Ruyang County in Luoyang city; Xichuan County, Xixia County, Neixiang County and Nanzhao County in Nanyang city; and Lushan County in Pingdingshan city. The Funiu Mountain water conservation area has a surface area of 24,058 km^2^, and the population is 5.3427 million (Source: Sanmenxia Statistical Yearbook, https://data.cnki.net/Search/ReportPreview?filename=N2020040335000047; Luoyang Statistical Yearbook accessed on 17 October 2021, https://data.cnki.net/Search/ReportPreview?filename=N2020050223000036 accessed on 17 October 2021; Nanyang Statistical Yearbook, https://data.cnki.net/Search/ReportPreview?filename=N2020040362000055 accessed on 17 October 2021; Pingdingshan Statistical Yearbook, https://data.cnki.net/Search/ReportPreview?filename=N2020090337000035 accessed on 17 October 2021).

### 2.2. Methods

#### 2.2.1. ESV Valuation of

The equivalent factor approach was first proposed by Costanza, an American ecologist, to evaluate the specific value of multiple service functions in an ecosystem [11]. Subsequently, Xie and other scholars [44] improved the classification system and value evaluation method for ecosystem services on this basis and obtained the value table of service functions in line with China’s terrestrial ecosystem. Because this method is simple to use, requires fewer data, and has high comparability of the evaluation results, it has become a commonly used method of ESV evaluation and has been adopted by most scholars [45].

(1) Equivalent correction of the ESV

Spatial heterogenous correction. When using the ecological service value table of China’s terrestrial ecosystem per unit area constructed by Xie et al. [44], considering that the value equivalent table is the average level of China as a whole, the table needs to be revised when it is being applied to the evaluation of the ESV in the Funiu Mountain water conservation area. The benchmark price of the ESV is the economic value of the annual natural grain yield of farmland under the condition of China’s average level. Therefore, our research revised the value equivalent using the grain yield in the study area to realize the scale transformation of the value equivalent table from the whole country to the Funiu Mountain area. One standard equivalent factor is equal to 1/7 of the market economic value of the annual grain yield per hectare of farmland. Our research used the economic value of grain production per unit area of farmland and the correction coefficient to modify the ESV equivalent factor. The formula for calculation is as follows [15]:

(1) $$E_{ij}=\alpha \frac{1}{7}\left(\frac{P\times Q}{A}\right)\times E_{0ij}$$

where $E_{ij}$ is the ecosystem service equivalent after regional correction; *P* is the average price of grain (yuan/kg); *Q* is the grain yield (kg); and *A* is the sown area of grain (hm^2^); (the grain in the Funiu Mountain area is mainly wheat, corn, and rice). *α* is the ESV correction coefficient of Henan Province, which is a fixed value, generally 1.39 [44]. $E_{0ij}$ is the ecosystem service equivalent in China, derived from the Chinese ESV equivalent table established by Xie et al. [44]. *i* = 1, 2, …, 5 represents the ecosystem types, which are cropland, forestland, grassland, water land, and unused land, respectively. *j* = 1, 2, …, 9 represents the ecosystem service types, such as food production service and raw material production service.

Eliminating the effects of inflation. When using the output value per unit area of grain in the study area for ESV equivalent correction, the price increase factor is not considered. As a result, the ESV time series difference in subsequent marginal change analysis does not have the same base. Therefore, even if the actual value of the ecosystem is the same, the value quantity represented by the price of the evaluation year will be different [46], which will directly affect the accuracy of the analysis results of marginal changes in the ESV. Therefore, considering that the research period spanned 15 years, to avoid the impact of inflation, based on the concept of comparable prices in economics, the consumer price index (CPI) was used to map the ESV of each period to the same price level to increase the comparability of the results. The formula for calculation is as follows [47]:

(2) $$VC_{ij}=E_{ij}\times \left(1+\frac{CPI^{\prime }-CPI_{0}}{CPI_{0}}\right)$$

where $VC_{ij}$ is the ecosystem service coefficient of the ESV in the Funiu Mountain area; $CPI^{\prime }$ is the fixed base CPI index; and $CPI_{0}$ is the current CPI index. In this way, our research can adjust the ESV in 2000, 2005, and 2010 to the price level in 2015, thus eliminating the interference of price fluctuations in value changes.

Based on Formulas (1) and (2), the ESV evaluation coefficient per unit area in the Funiu Mountain area in 2015 was finally obtained (Table 1).

(2) Calculation of the ESV

In this study, the geographical grid method was adopted to take each grid unit as the internal component granularity unit of regional ESV statistics, and ESV was calculated based on the statistics of the ESV of the land cover types in each grid. ESV grid calculation can not only express the spatial distribution and differentiation of the ESV but also facilitate the matching and fusion of multi-source spatial data for the coupling analysis of the ESV influencing factors of the ESV. Therefore, based on the scale of the research area and the requirements of ESV influencing mechanism analysis, this research used a 1 km × 1 km grid as the basic research unit to conduct ESV accounting in the Funiu Mountain area. The formula for calculation is as follows:

(3) $$ESV=\sum \left(A_{i}\times VC_{ij}\right)$$

where *ESV* is the ecosystem service value and $A_{i}$ is the area of ecosystem type *i*.

#### 2.2.2. Influencing Mechanism Analysis

(1) Determining the indicators that affect the ESV

The root of ESV changes is land use/land cover change (LUCC) [48,49,50]. LUCC affects the various functions of the ecosystem, and in doing so, it changes the value of ecosystem services and their temporal and spatial distribution patterns [51]. LUCC is the governance and transformation carried out by humankind based on the natural attributes of the land to achieve economic and social goals. This means that ecosystem services are closely related to the natural environment and socioeconomic development process. Therefore, the factors of ESV changes are often comprehensive.

Based on previous studies and the principles that can be spatially quantified [52,53], our research summarized the three major factors that influence the ESV from the perspective of the natural environment and human activities: natural factors, humanistic factors, and location factors. Natural factors are not only the basis for the existence of the ESV but also the constraints on the ESV. Although these factors are relatively stable in the short term, the difference in water, heat, and gas conditions caused by natural factors in the long term has an important impact on the spatial pattern of land use. Furthermore, these factors are inseparable from the formation and evolution of the distribution and structure of the ESV. Humanistic factors are the fundamental driving force of ESV changes and have a two-way causal relationship with the ESV. On the one hand, ensuring human well-being inevitably requires the ecosystem to provide more services. On the other hand, social and economic development is bound to change the original state of the ecosystem to varying degrees, which in turn restricts the supply of ecosystem services. The influence of location factors on the ESV is also very significant. The change in economic benefits caused by distance, which affects the key geographical elements of economic development (such as transportation and water systems), has a profound impact on regional land use patterns and affects the ESV [54]. Based on the analysis above, 15 influencing factors of the ESV were preliminarily drafted, including the topography, climate, soil texture, the normalized difference vegetation index (NDVI), economic development, the population level, the urbanization level, land location, traffic location, and water system location (Table 2).

(2) Random forest regression model

The RF algorithm is a machine learning method based on a classification tree proposed by Leo Breiman [55]. First, it uses the bootstrap resampling method to randomly select multiple samples from an original sample set with replacements to form a training sample. Second, feature vectors are randomly selected from the training sample data as candidate variables for decision tree splitting. Our research modelled a decision tree for each sample and then combined multiple decision trees to generate an RF. The final classification and prediction results were obtained by calculating the average value and majority voting [56]. The RF algorithm can handle high-dimensional data without feature selection. Its data type can be either discrete or continuous, and it can be used for clustering, discriminant, and regression analysis and can evaluate the importance of variables. In addition, due to the introduction of sample randomness and feature randomness, the RF algorithm has a certain anti-noise ability, and it does not easily fall into overfitting during operation. Based on these obvious and unique advantages, this study chose the RF algorithm to explore the influencing factors of the ESV.

## 2.3. Data Sources

The data used in this research are graphic image data and statistical data. The graph image data include land use data, vegetation coverage data, economic and population data, topography and geomorphology data, meteorological data, soil texture data, traffic data, and basic geographical data (Table 3). The statistical data came from the China County Statistical Yearbook and China Agricultural Products Price Survey Yearbook, which were mainly used to calculate the ESV.

## 3. Results

### 3.1. Spatiotemporal Variation in the ESV

#### 3.1.1. Characteristics of the Temporal Variation

The ESV in the Funiu Mountain area from 2000 to 2015 was calculated based on Formula (3). Table 4 shows that from the perspective of the first-level ecosystem service types, regulating services were the main service types provided by the Funiu Mountain area ecosystem, accounting for more than 50% of the total ESV. The rest were, from highest to lowest, supporting services, provisioning services, and cultural services. From the perspective of the secondary ecosystem service types, the order of the value of the ecosystem services from 2000 to 2015 remained relatively stable as follows: hydrological regulation > maintain biodiversity > soil conservation > gas regulation > climate regulation > waste treatment > raw material production > provide aesthetic landscape > food production. This result was mainly affected by the land use pattern dominated by woodland and grassland in the Funiu Mountain area.

From the perspective of temporal changes (Table 4), from 2000 to 2005, the ESV increased by 0.57%. This increase was mainly due to the implementation of the policy of returning farmland to forests in the Funiu Mountain area in 2002, which transferred sloped farmland and desertified farmland with severe soil erosion and low yield to forest or grassland with a higher ecological value. In the 2005–2010 and 2010–2015 periods, the ESV continued to decline, decreasing by 0.12% and 0.11%, respectively. This decline was caused by the process of rapid urbanization, in which the disorderly expansion of urban construction land gradually encroached on ecological land. From the perspective of different types of ecosystem services, the value of provisioning services and supporting services in the first-level category decreased by 2.00% and 0.41%, respectively; the value of regulating services and cultural services increased by 3.19% and 0.96%, respectively. Among the secondary types, the value of food production services decreased the most, with a reduction in value of 31 million yuan. In contrast, the value of waste treatment services improved the most, with an increase in value of 107 million yuan.

#### 3.1.2. Spatial Distribution and Variation Characteristics

**Figure 2:**
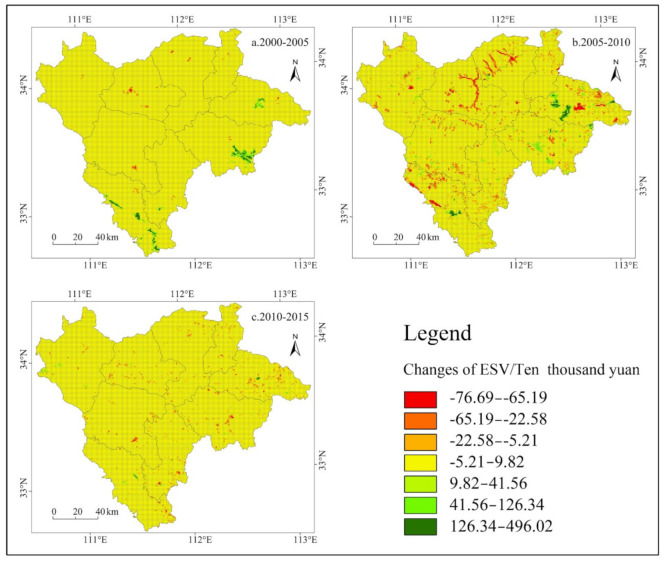
Spatial distribution of the ESV in different periods at the grid scale in the Funiu Mountain area.

Using the 1 km × 1 km grid as the basic unit (24,714 units in total), we calculated the ESV change in the Funiu Mountain area from 2000 to 2015. The natural breakpoint method can be used to set boundaries at relatively large numerical differences and to appropriately group similar values, and this approach can effectively characterize the characteristics of the spatial differentiation of the ESV [57]. Therefore, this research used the natural break point method to divide the change in the ESV in the Funiu Mountain area from 2000 to 2015 into 7 different intervals: (−76.69, −65.19], (−65.19, −22.58], (−22.58, −5.21], (−5.21, 9.82], (9.82, 41.56], (41.56, 126.34], and (126.34, 496.02] (Figure 2). Taking the county region as a unit, we evaluated the ESV and its change rate (Table 5) to analyze the law of the spatial differentiation rule of the ESV in the Funiu Mountain area.

Figure 2 and Table 5 shows that (1) the ESV changes in most regions of the Funiu Mountain area from 2000 to 2005 were small and were mainly concentrated in the interval (−5.21, 9.82). In contrast, the regions with faster ESV growth were distributed in the center of Lushan and Xichuan, southeast of Nanzhao, and south of Neixiang. Among them, Xichuan and Nanzhao had the largest increases in the ESV, with increases of 3.31% and 2.08%, respectively, and this result was mainly due to the improvement in ecosystem services that resulted from returning farmland to forests and the environmental management in the reservoir area. (2) From 2005 to 2010, the ESV declined on a large scale, especially in Luanchuan (decrease of 1.07%) and Songxian (decrease of 0.94%). The decrease in Songxian was mainly due to urban construction occupying ecological land. The decrease in Luanchuan was the result of the reduction in ecological land due to the combination of mining, tourism, and urbanization. In addition, due to the continuous implementation of the project of returning farmland to forest, the ESV of Lushan significantly increased. (3) From 2010 to 2015, except for the small increase in the ESV in Lushi due to the conversion of farmland to forests and the improvement in the ecological environment, the ESV of the other counties declined slowly. Among them, the southern areas of Xixia, the southern areas of Neixiang, and the central areas of Nanzhao had the most significant declines due to the expansion of the urban built-up area. 

### 3.2. Analysis of the Influencing Factors of the ESV

#### 3.2.1. Determining the RF Parameters

The RF algorithm has two very important parameters: mtry and ntree. mtry represents the number of feature vectors randomly selected from the original sample as candidate variables for the decision tree split, and ntree represents the number of decision trees. This research used out-of-bag (OOB) unbiased estimation to obtain the accuracy of the RF model under different parameters to set these two parameters [58]. MATLAB 2016b code (Windows-precompiled-RF_mexstandalone-v0.02, mex/standalone interface to Andy Liaw et al.’s C code, added by Abhi-shek Jaiantilal, Version: 0.02) was used to determine these two parameters.

**Figure 3:**
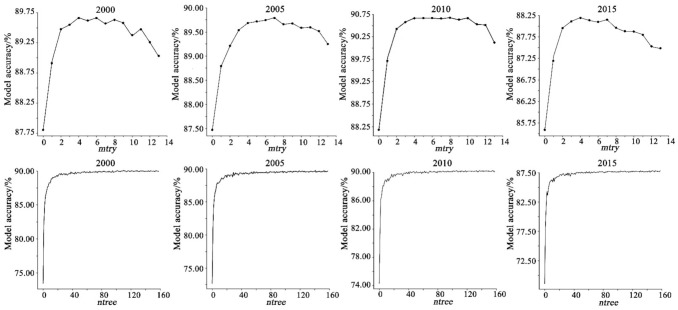
Relationship between RF model accuracy and mtry or ntree in the Funiu Mountain water conservation area.

First, in the case of a fixed ntree, our research tested the accuracy of the RF model under different mtry values. Our research took an ESV of 1 km × 1 km from 2000 to 2015 in the Funiu Mountain area as the basic data set, and the bootstrap sampling method was used to randomly select sample sets from these data sets with multiple replacements to form a new training sample set. To ensure the accuracy of the research results, the number of sample sets drawn each time must be large enough (usually 500), and 500 decision trees were constructed from these 500 sample sets. When sampling, some of the data were not selected (the proportion of data not selected was 36.8%). The data that were not selected constituted OOB data, which could be used to test the accuracy of the model (Figure 3). As shown in Figure 3, under the premise of a large number of decision trees (ntree = 500), the accuracy of the model decreased with an increase in mtry, with the highest accuracy at 4, 7, 10, and 4. Therefore, the mtry parameters in 2000, 2005, 2010, and 2015 were set to 4, 7, 10, and 4, respectively. Second, when mtry was set to 4, 7, 10, and 4, the accuracy of the RF model was tested for different ntree values (Figure 3). The accuracy of the model continued to improve with an increase in ntree, and the accuracy of the model was the highest and tended to be stable when ntree = 100. Considering model accuracy and computer performance comprehensively, the mtry and ntree parameter settings in 2000, 2005, 2010, and 2015 were (4, 100), (7, 100), (10, 100), and (4, 100), respectively. Finally, the set parameters were used to train the RF model based on the grid-scale ESV in the Funiu Mountain area to measure the importance of the influencing factors of the ESV.

#### 3.2.2. Coefficient of the Influencing Factors

Based on the RF model, the influence coefficients were obtained and graded. The lower the level was, the greater the influence of this factor on the ESV (Table 6). Table 6 shows that with the changes in the natural environment and socioeconomic conditions in the Funiu Mountain area, there were obvious differences in the degree of impact of different factors on the ESV.

First, our research analysed the first-level influencing factors. The influence coefficients of nature factors (X_1_–X_8_), humanities factors (X_9_–X_11_), and location factors (X_12_–X_15_) were 0.5708, 0.2190, and 0.2102 in 2000, 0.4806, 0.3783, and 0.1411 in 2005, 0.4284, 0.4581, and 0.1134 in 2010, and 0.4179, 0.5004, and 0.0817 in 2015, respectively. During the study period, the influence coefficient of natural factors decreased by 0.1529, while that of human factors and location factors increased by 0.2814 and 0.1285 respectively. This phenomenon reveals that although natural factors serve as the substrate and background for the formation of ecosystem services, they are important factors that affect the ESV in the Funiu Mountain area in the long term. However, since the beginning of the 21st century, human activities have caused changes in land use patterns, continuous improvements in economic development, and population agglomeration, which have affected the habitat quality and ecosystem structure of the Funiu Mountain area. The impact of relatively fast-changing humanistic factors has gradually surpassed that of relatively stable natural factors, and humanistic factors have become the dominant driving factor affecting the ESV in the Funiu Mountain area. Second, our research analysed the third-level influencing factors. In 2000, the distribution of the ESV in the Funiu Mountain area was not dominated by a significant single driving factor. Relatively important influencing factors included the distance from rural settlements, population density, precipitation, elevation, the NDVI, and slope. From 2005 to 2015, in addition to the large influence coefficient of the NDVI and precipitation, population density became the dominant driving factor of the ESV.

The change in the coefficient of each influencing factor mainly showed the following characteristics. First, population density, the NDVI, economic density, the distance from urban areas, the urbanization level, the distance from major water systems, and the distance from major roads had increasing impacts on the ESV. Among them, the urbanization level, population density, the distance from main roads, and economic density increased rapidly, and their influence coefficients increased by 154%, 142%, 98%, and 74%, respectively. Population density and economic density currently have a greater impact on the distribution of the ESV in the Funiu Mountain area. The influence coefficient of population density reached 0.3876 in 2015 and has since become the most important influencing factor. Notably, the influence coefficients of the urbanization level and distance from major roads in 2015 were only 0.0318 and 0.0036, respectively, and they are not currently the main factors affecting the ESV. However, judging from the continuous and rapid growth in their influence coefficients, their influence on the ESV will greatly increase in the future. Second, the impacts of precipitation, the distance from rural settlements, elevation, slope, temperature, soil sand content, soil clay content, and soil silt content on the ESV decreased. Among them, the fastest decline was the distance from rural settlements; its influence coefficient dropped from 0.1890 to 0.0480, a decrease of 75%, and it went from being the most important influencing factor in 2000 to the fifth most important influencing factor in 2015. The next influencing factors were slope and elevation; their degrees of influence decreased by 73% and 68%, respectively. However, even if the impact of these two factors weakened, they still had a certain control effect on the ESV as the basis for the existence of the ecosystem and the functioning of ecological functions.

#### 3.2.3. Gradient Differentiation of the Influencing Factors

Gradient analysis is an effective method of quantitatively identifying the gradient or regular changes in the ESV driven by various influencing factors [59,60]. This research used ArcGIS spatial analysis tools to select seven relatively important ESV driving factors, i.e., slope, elevation, precipitation, the NDVI, population density, economic density, and distance from rural settlements, to explore the law of the differentiation of the ESV under different gradient levels.

**Figure 4:**
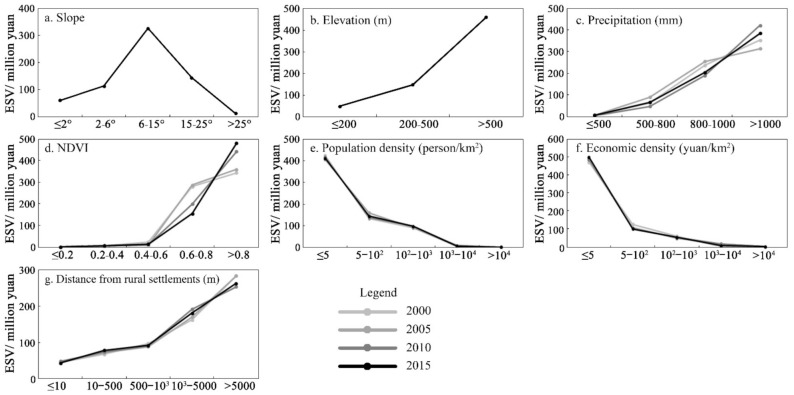
ESV distribution under different gradients of influencing factors in the Funiu Mountain area.

**Figure 5:**
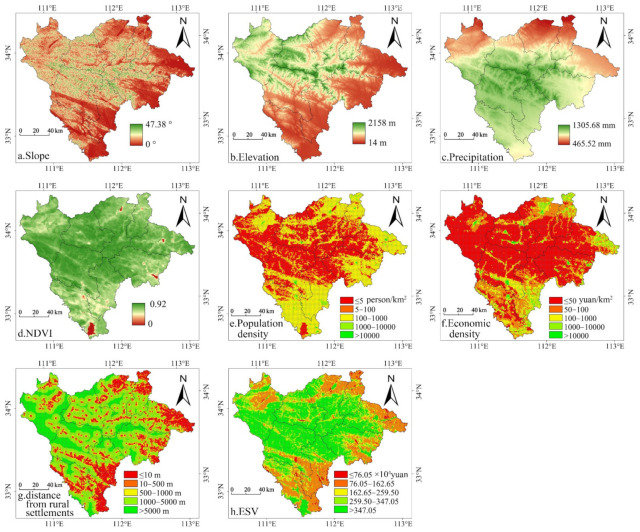
The spatial distribution pattern of different influencing factors of the ESV in the Funiu Mountain area in 2015.

Based on the classification requirements specified in the Technical Regulations for the Third National Land Survey issued by the Ministry of Natural Resources of China and the topographic features of the Funiu Mountain area, slope was divided into five levels: flat (≤2°), gentle slope (2–6°), slope (6–15°), steep slope (15–25°), and dangerous slope (>25°); elevation was divided into three levels: plains (≤200 m), hills (200~500 m), and mountains (>500 m). Following previous research results [61,62], we divided the NDVI into five levels based on the equidistant method: low coverage (≤0.2), medium and low coverage (0.2–0.4), medium coverage (0.4–0.6), medium to high coverage (0.6–0.8), and high coverage (>0.8). The four factors of precipitation, population density, economic density, and the distance from rural settlements were classified using the natural break point method in ArcGIS. Precipitation was divided into four levels: ≤500 mm, 500–800 mm, 800–1000 mm, and >1000 mm. Population density was divided into five levels: ≤5 persons/km^2^, 5–100 persons/km^2^, 100–1000 persons/km^2^, 1000–10,000 persons/km^2^, and >10,000 persons/km^2^. Economic density was divided into five levels: ≤50 yuan/km^2^, 50–100 yuan/km^2^, 100–1000 yuan/km^2^, 1000–10,000 yuan/km^2^, and >10,000 yuan/km^2^. Additionally, the distance from rural settlements was divided into five levels: ≤10 m, 10–500 m, 500–1000 m, 1000–5000 m, and >5000 m. According to the grading standards above, the ESV of each factor at different gradient levels from 2000 to 2015 was quantified (Figure 4). Combined with the spatial distribution pattern of various factors in 2015 (Figure 5), the law of the gradient differentiation of ESV influencing factors in the Funiu Mountain area was y analysed in depth.

Topography (Elevation and Slope). Regarding elevation, from 2000 to 2015, there was a positive correlation between the ESV and elevation in the Funiu Mountain area. That is, the ESV increased with elevation, showing obvious vertical differentiation characteristics (Figure 4a and Figure 5a). Mountainous areas with elevations greater than 500 m account for approximately 70% of the ESV. Hilly areas with elevations of 200 to 500 m account for approximately 23% of the ESV. Plains areas with elevations below 200 m account for approximately 7% of the ESV. Regarding slope, based on the changes in the ESV at different slope grades (Figure 4b and Figure 5b), the ESV of the area below 15° increased with increasing slope, and the area above 15° decreased with increasing slope.

Precipitation. The most significant climatic factor affecting the ESV in the Funiu Mountain area from 2000 to 2015 was precipitation. Figure 4c and Figure 5c show that the ESV and precipitation had a significant positive correlation and that the ESV increased with increasing precipitation. From 2000 to 2015, the ESV of the area with an annual precipitation less than 500 mm was low, accounting for only 0.49%, 0.53%, 0.41%, and 0.75% of the total value; the ESV of the area with an annual precipitation above 1400 mm was higher, accounting for 53.91%, 47.57%, 63.96%, and 58.55% of the total value.

NDVI. The correlation coefficients between the NDVI and ESV from 2000 to 2015 were 0.1085, 0.1154, 0.1539, and 0.1701, indicating a significant positive correlation between them. That is, the ESV increased with increasing vegetation coverage. In 2015, the proportion of the ESV in areas with high vegetation coverage reached 73.08%, while that in areas with low vegetation coverage was only 0.12% (Figure 4d and Figure 5d).

Population. The population density and ESV in the Funiu Mountain area showed a strong negative correlation, indicating that the lower the population density was, the higher the ESV was (Figure 4e and Figure 5e). The ESV of areas with a population density of less than 5 persons/km^2^ accounted for more than 60% of the total value, while areas with a population density of more than 100 persons/km^2^ accounted for only 15% of the total ESV.

Economic development. There was a negative correlation between the ESV and economic density in the Funiu Mountain area. That is, the greater the economic density was, the smaller the ESV was (Figure 4f and Figure 5f). Among them, more than 70% of the ESV was concentrated in areas where the economic density was less than 50 yuan/km^2^.

Distance from rural settlements. From 2000 to 2015, among the location factors, the distance from rural settlements had the most significant impact on the ESV, and the two were positively correlated. That is, the ESV was higher with an increasing distance from rural settlements. Figure 4g and Figure 5g show that areas with a high ESV were mostly concentrated in areas 2000 m from rural settlements, with values of 446.84, 454.36, 44.63, and 44.471 billion yuan, with the proportions reaching 68.19%, 68.94%, 67.85%, and 67.64%, respectively.

## 4. Discussion

### 4.1. Methods

#### 4.1.1. ESV Evaluation Method

ESV evaluation methods have their own advantages and disadvantages. The equivalent factor method selected in this research has the advantages of simple calculation and high comparability and can provide convenience for large-area, long-term ESV evaluation. However, due to the complexity, dynamics and nonlinear characteristics of the ecosystem, the assessment results have certain variability and uncertainty. For example, construction land may have a negative impact on the ecosystem. However, a consensus on the value coefficient of construction land has yet to be reached, and in this study, the proportion of construction land area was negligible due to the huge regional area. For these reasons, this research did not calculate its ecological value, and as a result, the obtained ESV may have a certain deviation.

Nevertheless, the equivalent factor method has been successfully applied in many research cases [63,64]. The use of time series instead of time cross-section analysis can reduce or offset the uncertainty and errors in the evaluation of the equivalent factor method. Furthermore, the sensitivity analysis conducted by Liu et al. [65] showed that although the value coefficient of the equivalent factor method exhibited uncertainty, the evaluation results were still robust. Therefore, although the value coefficient of the equivalent factor method is relatively biased, the spatiotemporal changes in the ESV that it reflects still have practical significance. 

In addition, due to data limitations, this research failed to use the net profit per unit area of grain crops in different counties (districts) to approximately replace the value of 1 standard unit ESV equivalent factor. Only the introduction of the CPI corrects the ESV coefficient per unit area. Therefore, more accurate ESV estimation methods and more detailed index data should be used in future studies to improve the accuracy of ESV calculations and ensure the reliability of ESV data.

#### 4.1.2. ESV Influencing Factor Analysis Method

Ecosystem services are an enormous and complex system of land, the social economy, and ecology. Thus, the ESV is complex, nonstructural, and random, which requires more robust and flexible methods to deal with nonlinear relationships, high-order correlations, and even missing values. Meanwhile, over time and space, the degree of influence of various factors on the ESV may change, and the initial weight may not conform to the actual situation, further promoting the development of a measurement model for nonparametric values. As a nonparametric tree model, the RF model can effectively solve the problems above and become an effective tool for research on the influencing factors of the ESV.

**Figure 6:**
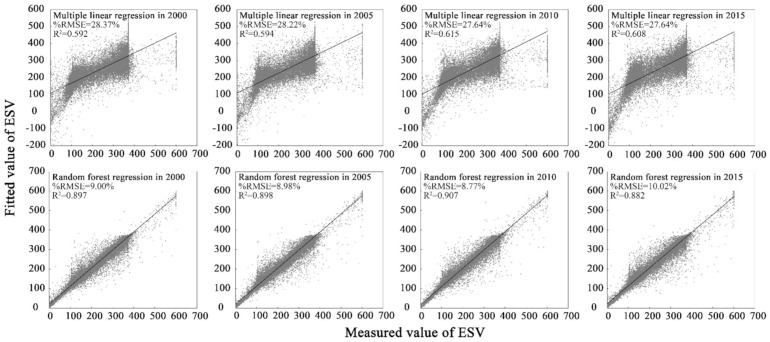
Scatter plot of ESV verification based on multiple linear and RF regression in the Funiu Mountain area.

Using 15 influencing factors as independent variables, we performed multiple linear regression and RF regression on the ESV, calculated the ESV simulation value, coefficient of determination (R^2^), and relative root mean square error (%RMSE), and used the actual ESV value and the simulated value to generate a scatter plot (Figure 6). The R^2^ values of the multiple linear regressions were 0.592, 0.594, 0.615, and 0.608, which were much smaller than the RF-based R^2^ values (0.897, 0.898, 0.907, and 0.882). The %RMSE of the multiple linear regression was 28.37%, 28.22%, 27.64%, and 27.64%, indicating that its simulation accuracy reached 71.63%, 71.78%, 72.36%, and 72.36%, which was much smaller than the simulation accuracy of the RF regression (91.00%, 91.02%, 91.23%, 89.98%). Compared with the multiple linear regression, the ESV measured value and the simulated value under the RF algorithm had a more obvious linear fitting relationship, and there were negative values in the ESV-simulated value of the multiple linear regression, contradicting the actual meaning of the ESV in this research. Therefore, the RF algorithm applied to research the influencing factors of the ESV under the background of big data had higher interpretative accuracy than the traditional multiple linear regression model.

Previous studies also provide support for the high precision and high accuracy of the RF model. Zhang et al. [66] used massive data from atmospheric monitoring stations to identify the main pollutant sources of PM2.5 in urban air in Wuhan city based on the RF model, and the results showed that the R^2^ of the RF model reached more than 0.85. Huang et al. [67] used the RF model to explore the sources of soil heavy metals and found that the interpretative accuracy of the RF model reached more than 75%. Some scholars have compared the RF model with other models, such as Xu et al. [68], who studied the key elements of the soil-forming environment, and Chen et al. [69], who studied the influencing factors of cultivated land utilization efficiency. Both found that the accuracy of the RF model was significantly higher than that of the artificial neural network model.

However, the RF model constructed in this research mainly considers the influence of deterministic factors on the ESV, which is only a preliminary simplified model. In addition, the weights of each factor are automatically obtained in the training process based on the laws of the evolution of real data, which is not applicable for the prediction of the ESV in the future. In combination with actual social and economic development, further improving the model to improve its evaluation performance will be the focus and challenge of subsequent research.

Finally, because the influencing factors of the ESV are complex and nonlinear, even if all factors are included in the ESV assessment system, there may still be certain uncertainties in the results. In addition, because the RF algorithm does not consider the spatial nonstationary characteristics of the data, it cannot quantify the spatial heterogeneity of the influencing factors of the ESV. Future work should focus on the difference in the interpretative accuracy of the influencing factors of the ESV by using different attribution methods (such as neural networks) to improve the accuracy of the results. Furthermore, considering the differences between the regional natural environment and cultural environment, the relationship between influencing factors will change with changes in spatial location. Therefore, machine learning methods and spatial analysis methods (such as geographically weighted regression) can be organically combined to explore the spatial heterogeneity of the influencing factors of the ESV. Finally, policy factors such as returning farmland to forests, land consolidation, regional economic regulation, and ecological compensation have a certain degree of impact on the ESV. However, due to the difficulty of data acquisition and quantification, policy factors were not considered in this study. Especially for the Funiu Mountain area, which spans four prefectures and cities, the next step in conducting research should be to integrate policy differences and discuss how to incorporate policy factors into the ESV influencing factor analysis system.

### 4.2. Influencing Factors of the ESV

According to previous studies [40,70], changes in the ESV are the result of the joint action of natural factors and humanistic factors, and the ESV is mainly affected by humanistic factors in a short period of time, which is consistent with the research conclusion of this article.

First, the ESV in the Funiu Mountain area presents significant spatial differentiation, mainly due to the following reasons: the limitation effect of natural environmental conditions and the stress effect caused by human activities. 

In terms of natural environmental conditions, there is a large area of hills and mountains in the western Funiu Mountain area. Complex terrain conditions cause human activities and the ESV to show significant characteristics of differentiation. This phenomenon is mainly due to the high elevation and steep slope of the region with a high topographic position index, as well as a variety of environmental factors, such as inappropriate climate conditions, which restrict human production activities, development, and construction. Therefore, the natural ecosystem on the higher terrain gradient is disturbed and damaged to a low degree, which may be the reason why ESV of mountainous regions is generally higher than that of plains regions. In terms of human activities, the natural resources in the eastern Funiu Mountain area are rich, leading to the development and utilization of various natural resources and the destruction of the local natural environment for urban construction and arable land expansion. In this process, a series of environmental pollution problems, such as water pollution and air pollution, occur. Therefore, human activities have an obvious stress effect on the natural ecological environment. These phenomena are mostly concentrated in flat terrain areas, and the more densely populated the areas are, the more significant the stress effect.

Second, the main influencing factors (elevation, slope, precipitation, the NDVI, population, economic development, the distance from rural settlements) of the ESV changes in the Funiu Mountain area are discussed in detail.

Elevation affects the spatial pattern of the ESV through the distribution of land types and the range and intensity of human activities in the Funiu Mountain area. There were more restrictions on human activities at higher elevations, and the land use types were mainly woodland and high-coverage grassland, with relatively stable ecological structure and a higher ESV. The middle elevation areas had frequent human activities. The land use types were mainly cultivated land and low-to-medium coverage grassland, and the ESV was lower. The dominant land use types in low elevation areas were cultivated land and construction land. Although there were some water areas, they accounted for a small proportion and provided insignificant ecological functions. Therefore, the ESV in such areas was the lowest.

Slope makes it difficult for humans to use land and affects the structure and function of ecosystems through the formation of a heterogeneous environment in a local area, thereby affecting the ESV. The impact of slope on the ESV in the Funiu Mountain area is extremely complex. The ESV at different slope grades not only was affected by the land use type but also depended on the amount of land area in the slope grade to a certain extent. Although the dominant land types with slopes above 15° in the Funiu Mountain area were woodland and grassland with a higher ecological value, the ESV was only 23.81% of the total value due to the small proportion of their area (27.55%). In the area between 6° and 15° of slope, although the land types were diverse and the land structure was complex, there was not only ecological land such as woodland and grassland but also land for human production and living such as cultivated land and construction land. However, it covered a large area (50.72%), becoming the area with the highest ESV, accounting for 49.76% of the total value. 

An increase in precipitation can promote the growth of vegetation, especially natural forests, is conducive to the production of organic matter and the accumulation of nutrients, and can improve regional water conservation, soil conservation, and climate regulation, thereby increasing the ESV. Therefore, in the Funiu Mountain area, the area with more precipitation has the highest ESV.

In the Funiu Mountain area, most areas with higher vegetation coverage are designated nature reserves, which are natural ecosystems with complete functions and basically no human activities. Thus, these areas have strong ecosystem regulating and supporting functions. With the decline in vegetation coverage, the traces of human activities have become more obvious, and the disturbance to the ecosystem has gradually increased. Especially in areas with low vegetation coverage, the terrain is flat, transportation is convenient, the type of land is mainly construction land, and the ecosystem functions are weak.

Population agglomeration is an inevitable result of the process of urbanization. As the urbanization rate increases, the population gradually migrates from rural to urban areas. From 2000 to 2015, the urban population density in the Funiu Mountain area increased from 614,300 to 886,600 persons per square kilometer, for a growth rate of 44.33%. Population growth caused urban construction land to occupy a large amount of ecological land, resulting in a decrease in the total ESV of 11,476,100 yuan, which represents a decrease of 36.84%. Population agglomeration increases the total resource consumption of cities and towns as well as the discharge of urban waste. If the urban population can be kept within the tolerance threshold of the ecosystem, then humans and nature can remain coordinated and develop sustainably. Once it exceeds the carrying capacity of the ecosystem, great pressure will be placed on the self-regulation function of the ecosystem, and irreversible damage will be caused to the ecological environment.

During the study period, the GDP of the Funiu Mountain area increased from 17.376 billion yuan to 133.061 billion yuan, an increase of 6.66 times. Clearly, the increase in economic activities and the increase in economic output caused a gradual reduction in forestland, grassland, and arable land. Furthermore, the continuous expansion of urban, industrial, and transportation lands caused profound changes in the original ecosystem and affected the supply of ecosystem services. With the strategic demands of sustainable development and ecological civilization construction, coordinated economic and ecological development has become an important issue in the future development of the Funiu Mountain area. Whether it continues the strategy of considering ecological environmental protection under the goal of economic maximization or pursues the maximization of the ESV on the basis of ensuring certain economic benefits from growth [71], the local government needs to think deeply and make prudent decisions.

Considering the characteristics of the topography and geomorphology of the Funiu Mountain area, rural settlements are generally distributed in relatively flat areas. To meet the needs of production and life, the transformation of surrounding land is strong. However, with increasing distance from rural settlements, the weaker the impact of human activities is, the stronger the originality of the ecosystem, the better the functions, and the higher the ESV.

## 5. Conclusions

Taking the Funiu Mountain water conservation area in China as an example, this work revealed the spatio-temporal patterns of the ESV by using the revised value equivalent factor method and geographical information system (GIS) spatial analysis method, and it quantitatively studied the influencing factors of the ESV by using an RF model from the comprehensive perspective of the natural environment and human activities. The main conclusions were as follows:

(1) The ESV first increased and then decreased, increasing by 375 million yuan from 2000 to 2005 and decreasing by 154 million yuan from 2005 to 2015. In 2015, the ESV reached 65.754 billion yuan, an increase of 0.34% compared with 2000. The spatial characteristics of the variation in the ESV were obvious. The areas showing increases were mainly distributed in Lushan, Nanzhao, and Xichuan, and those showing decreases were mainly distributed in Luanchuan, Songxian, Ruyang and Xixia. (2) Among the first-level service types, regulating services accounted for a large proportion. Among the second-level service types, hydrological regulation, biodiversity maintenance, soil conservation, gas regulation, and climate regulation services accounted for a large proportion. (3) The ESV in the Funiu Mountain water conservation area was influenced by the comprehensive effects of the natural environment and human activities. Overall, the natural environment played a fundamental role in controlling the spatial distribution of the ESV. The intensity of human activities gradually increased, leading to a change in the direction of the ESV. (4) The influence coefficient of natural factors decreased by 0.1529, and the humanistic factors and location factors increased by 0.2814 and 0.1285, respectively. With economic development, the effect of human activities on the ESV may be further intensified. (5) The NDVI was the most positive influencing factor, followed by precipitation. Population density was the most negative influencing factor, followed by economic density. Although the influence coefficient of the urbanization level was only 0.0318 in 2015, it increased by 154.41% compared with 2000. Our research concludes that the influence of the urbanization level will greatly increase in the future.

Based on the characteristics of the temporal and spatial changes in the ESV, the areas where the ESV decreased significantly during the study period were concentrated in urban built-up areas. These areas have flat terrain, a dense population, intense economic activities, and a strong development intensity. The ecological management of these areas should start from two aspects. For cities, it is necessary to make full use of spot space and to improve urban green space facilities. When it is impossible to considerably increase the green space system, the accessibility of urban space and the green space system should be improved to realize the full development of green space. For the areas surrounding cities, overall planning and reasonable layouts should be developed to increase the compactness of industrial land and to improve land use efficiency.

There are a large number of river systems and large medium-sized reservoirs in Xichuan County. The Danjiangkou Reservoir is the water source for the Middle Route Project of China’s South-to-North Water Diversion Project, and it is an important ecological barrier for Henan Province and even for China as a whole. Although the ESV of Xichuan County increased during the study period, its change trend showed a state of first increasing and then decreasing. This result shows that effective progress has been made in ecological conservation in recent years, but due to the continuous expansion of the scale of towns and the increase in population, there has still been a certain negative impact on the ecosystem. In the future, ecological civilization construction should be prioritized. The core task is to continue to intensify the comprehensive management of the ecological environment of the reservoir, increase the intensity of closing hills for afforestation, and return farmland to forests. Considering the actual situation of Xichuan County, the development of ecological tourism should be prioritized. The impact of high-energy-consuming secondary industries on the environment should be minimized to achieve stability and to improve the regional environment.

Luanchuan County and Song County are the main tourist counties in the Funiu Mountain area, and the ESV decreased significantly during the study period. In recent years, due to the vigorous development of the tourism industry, local resources have been greatly depleted and wasted. While attracting tourism revenue, the development of this industry has also increased environmental pollution and destruction and caused irreversible damage to the natural environment and resources. Therefore, ecological management should be carried out in three ways. First, the overall principle should focus on protection, supplemented by development, and existing mountains and water sources should be protected. Carrying out various development activities should be strictly forbidden, and at the same time, the prevention and management of forest fires and pests should be strengthened. Second, an ecological compensation mechanism should be adopted for core protected areas to encourage residents to relocate and reduce their ecological load. Finally, measures should be adapted to local conditions, and biological resources should be utilized to develop green, high-efficiency, and pollution-free industries. Natural resources such as beautiful landscapes, forests, fields, and lakes should be used to develop characteristic ecotourism based on the characteristics of local resources. Economic benefits should improve within the ecological capacity to ensure the stability of the ecosystem.

## Figures and Tables

**Table 1:** Coefficient of the ESV in the Funiu Mountain area in 2015.

First Category	Second Category	Cropland	Forestland	Grassland	Water Land	Unused Land
Provisioning services	Food production	1324.5	437.1	569.5	702.0	26.5
	Raw material production	516.5	3947.0	476.8	463.6	53.0
Regulating services	Gas regulation	953.6	5721.8	1986.7	675.5	79.5
	Climate regulation	1284.8	5390.7	2066.2	2728.4	172.2
	Hydrological regulation	1019.9	5417.1	2013.2	24,860.6	92.7
	Waste treatment	1841.0	2278.1	1748.3	19,668.6	344.4
Supporting services	Soil conservation	1947.0	5324.4	2966.8	543.0	225.2
	Maintain biodiversity	1351.0	5973.4	2476.8	4543.0	529.8
Cultural services	Provide aesthetic landscape	225.2	2754.9	1152.3	5880.7	317.9

**Table 2:** Influencing factors of the ESV.

First-Level Influencing Factors	Second-Level Influencing Factors	Third-Level Influencing Factors
Natural factors	Topography	Elevation (X_1_)
		Slope (X_2_)
	Climate	Temperature (X_3_)
		Precipitation (X_4_)
	Soil texture	Silt content of soil (X_5_)
		Clay content of soil (X_6_)
		Sand content of soil (X_7_)
	Vegetation coverage	NDVI (X_8_)
Humanistic factors	Economic development	Economic density (X_9_)
	Population	Population density (X_10_)
	Urbanization	Land urbanization level (X_11_)
Location factors	Land location	Distance from urban area (X_12_)
		Distance from rural settlements (X_13_)
	Traffic location	Distance from main traffic road (X_14_)
	Water location	Distance from main river system (X_15_)

**Table 3:** Sources of graphic image data.

Data Type	Description	Source
Land use Data	The land use data were generated through artificial visual interpretation based on Landsat TM/ETM images of 2000, 2005, 2010, and 2015. The resolution of these images is 30 m. Finally, we obtained six land use types: cultivated land, forest land, grassland, water area, construction land, and unused land.	U.S. Geological Survey (https://glovis.usgs.gov/ accessed on 17 October 2021)
Vegetation Coverage Data	The vegetation coverage data were from MODND1T China’s 500 m NDVI composite products and resampled to 1 km × 1 km.	Geospatial Data Cloud (http://www.gscloud.cn/ accessed on 17 October 2021)
Economic and Population Data	The GDP and population data of the county unit were converted to a scale of 1 km × 1 km by combining the data of land use, night light, and residential density.	Resource and Environment Data Cloud Platform (http://www.resdc.cn/ accessed on 17 October 2021)
Topography and Geomorphology Data	The elevation and slope were extracted from DEM data with a resolution of 250 m and then resampled to 1 km × 1 km.	Geospatial Data Cloud (http://www.gscloud.cn/ accessed on 17 October 2021)
Meteorological Data	Based on the daily temperature and precipitation data from meteorological stations from 2000 to 2015, the spatial interpolation generated the precipitation and annual average temperature data of 1 km × 1 km.	China Meteorological Data Sharing Service (http://data.cma.cn/ accessed on 17 October 2021)
Soil Texture Data	Data were generated according to the soil type map and the second soil Census data, including soil silt content, soil clay content, and soil sand content.	Resource and Environment Data Cloud Platform (http://www.resdc.cn/ accessed on 17 October 2021)
Traffic Data	Combined with the map and planning drawings, the traffic data from 2000 to 2015 were sorted out, and only the main roads such as railway, high-speed, provincial road, county road, and township road were retained.	Open Street Map (https://www.openstreetmap.org/ accessed on 17 October 2021)
Basic Geographic Data	Data were generated according to the navigation database, Google earth, and some measured data, including county and township administrative division data.	Data Center of Lower Yellow River Regions (http://henu.geodata.cn accessed on 17 October 2021)

**Table 4:** Changes in the structure of the ESV in the Funiu Mountain area from 2000 to 2015.

Types of Ecosystem Services		ESV/Billion Yuan				Rate of Change/%		
First Category	Second Category	2000	2005	2010	2015	2000–2005	2005–2010	2010–2015
Provisioning services	Food production	1.71	1.70	1.69	1.68	−0.47	−0.77	−0.60
	Raw material production	5.87	5.86	5.86	5.86	−0.05	0.00	−0.11
Regulating services	Gas regulation	8.95	8.95	8.95	8.94	−0.05	0.01	−0.14
	Climate regulation	8.89	8.90	8.90	8.88	0.10	−0.05	−0.15
	Hydrological regulation	10.18	10.34	10.35	10.35	1.86	−0.20	0.05
	Waste treatment	6.10	6.23	6.20	6.20	2.29	−0.48	−0.04
Supporting services	Soil conservation	9.34	9.32	9.32	9.30	−0.16	−0.08	−0.22
	Maintain biodiversity	9.95	9.97	9.96	9.95	0.23	−0.05	−0.13
Cultural services	Provide aesthetic landscape	4.56	4.60	4.60	4.60	1.00	−0.03	0.00
Total		65.53	65.91	65.83	65.75	0.57	−0.12	−0.11

**Table 5:** ESV and ESV change rate in the Funiu Mountain area from 2000 to 2015.

County	ESV/Billion Yuan				Rate of Change/%		
	2000	2005	2010	2015	2000–2005	2005–2010	2010–2015
Lushi	10.307	10.306	10.281	10.307	−0.01	−0.24	0.25
Lushan	5.484	5.523	5.636	5.623	0.71	2.06	−0.24
Luanchuan	8.192	8.177	8.090	8.077	−0.18	−1.07	−0.15
Nanzhao	7.924	8.089	8.135	8.116	2.08	0.58	−0.23
Neixiang	5.290	5.295	5.301	5.289	0.09	0.11	−0.23
Ruyang	3.162	3.160	3.150	3.143	−0.08	−0.31	−0.22
Songxian	8.575	8.577	8.496	8.485	0.02	−0.94	−0.14
Xixia	10.900	10.895	10.872	10.852	−0.05	−0.20	−0.19
Xichuan	5.699	5.887	5.865	5.862	3.31	−0.38	−0.04

**Table 6:** Importance grading of ESV influencing factors in the Funiu Mountain area.

Grade	Influence Coefficient	2000	2005	2010	2015
Level 1	>0.3	No data	X_10_	X_10_	X_10_
Level 2	0.15~0.3	X_13_, X_10_, X_4_	X_4_	X_8_	X_8_
Level 3	0.05~0.15	X_1_, X_8_, X_2_	X_13_, X_8_, X_1_, X_2_	X_4_, X_9_, X_13_	X_4_, X_9_
Level 4	0.01~0.05	X_9_, X_3_, X_7_, X_12_, X_11_, X_6_, X_5_	X_9_, X_7_, X_3_, X_11_, X_12_, X_6_	X_1_, X_2_, X_12_, X_11_, X_7_, X_3_	X_13_, X_1_, X_2_, X_11_, X_3_, X_12_, X_7_
Level 5	≤0.01	X_14_, X_15_	X_5_, X_15_, X_14_	X_15_, X_5_, X_6_, X_14_	X_15_, X_5_, X_6_, X_14_

## References

[1] Costanza R., De Groot R., Sutton P., Van Der Ploeg S., Anderson S.J., Kubiszewski I., Farber S., Turner R.K. (2014). Changes in the global value of ecosystem services. Glob. Environ. Chang..

[2] Brauman K.A., Daily G.C., Duarte T.K., Mooney H.A. (2007). The nature and value of ecosystem services: An overview highlighting hydrologic services. Annu. Rev. Environ. Resour..

[3] Serafy S. (1998). Pricing the invaluable: The value of the world’s ecosystem services and natural capital. Ecol. Econ..

[4] Braat L.C., de Groot R. (2012). The ecosystem services agenda: Bridging the worlds of natural science and economics, conservation and development, and public and private policy. Ecosyst. Serv..

[5] Delgado L.E., Marín V.H. (2020). Ecosystem services and ecosystem degradation: Environmentalist’s expectation?. Ecosyst. Serv..

[6] Millennium Ecosystem Assessment (2005). Ecosystems and Human Well-Being: Synthesis.

[7] De Groot R., Brander L., Van Der Ploeg S., Costanza R., Bernard F., Braat L., Christie M., Crossman N., Ghermandi A., Hein L. (2012). Global estimates of the value of ecosystems and their services in monetary units. Ecosyst. Serv..

[8] Dai X., Johnson B.A., Luo P., Yang K., Dong L., Wang Q., Liu C., Li N., Lu H., Ma L. (2021). Estimation of urban ecosystem services value: A case study of Chengdu, Southwestern China. Remote Sens..

[9] Yin D., Li X., Li G., Zhang J., Yu H. (2020). Spatio-temporal evolution of land use transition and its eco-environmental effects: A case study of the Yellow River Basin, China. Land.

[10] Benayas J.M.R., Newton A.C., Diaz A., Bullock M.J. (2009). Enhancement of biodiversity and ecosystem services by ecological restoration: A meta-analysis. Science.

[11] Costanza R., D’Arge R., de Groot R., Farber S., Grasso M., Hannon B., Limburg K., Naeem S., O’Neill R.V., Paruelo J. (1997). The value of the world’s ecosystem services and natural capital. Nature.

[12] Seppelt R., Fath B., Burkhard B., Fisher J., Grêt-Regamey A., Lautenbach S., Pert P., Hotes S., Spangenberg J., Verburg P. (2012). Form follows function? Proposing a blueprint for ecosystem service assessments based on reviews and case studies. Ecol. Indic..

[13] Burkhard B., Kroll F., Nedkov S., Müller F. (2012). Mapping ecosystem service supply, demand and budgets. Ecol. Indic..

[14] Ghermandi A., Fichtman E. (2015). Cultural ecosystem services of multifunctional constructed treatment wetlands and waste stabilization ponds: Time to enter the mainstream?. Ecol. Eng..

[15] Xie G., Zhang C., Zhen L., Zhang L. (2017). Dynamic changes in the value of China’s ecosystem services. Ecosyst. Serv..

[16] Guo A., Zhang Y., Zhong F., Jiang D. (2020). Spatiotemporal patterns of ecosystem service value changes and their coordination with economic development: A case study of the Yellow River Basin, China. Int. J. Environ. Res. Public Health.

[17] Song W., Deng X.Z., Yuan Y.W., Wang Z., Li Z.H. (2015). Impacts of land-use change on valued ecosystem service in rapidly urbanized North China Plain. Ecol. Model..

[18] Tianhong L., Wenkai L., Zhenghan Q. (2010). Variations in ecosystem service value in response to land use changes in Shenzhen. Ecol. Econ..

[19] Chee Y.E. (2004). An ecological perspective on the valuation of ecosystem services. Biol. Conserv..

[20] De Groot R.S., Alkemade R., Braat L., Hein L., Willemen L. (2010). Challenges in integrating the concept of ecosystem services and values in landscape planning, management and decision making. Ecol. Complex..

[21] Roces-Díaz J.V., Díaz-Varela R.A., Álvarez-Álvarez P., Recondo C., Díaz-Varela E.R. (2015). A multiscale analysis of ecosystem services supply in the NW Iberian Peninsula from a functional perspective. Ecol. Indic..

[22] Richardson L., Loomis J., Kroeger T., Casey F. (2015). The role of benefit transfer in ecosystem service valuation. Ecol. Econ..

[23] Zhao B., Kreuter U., Li B., Ma Z., Chen J., Nakagoshi N. (2004). An ecosystem service value assessment of land-use change on Chongming Island, China. Land Use Policy.

[24] Fu B., Li Y., Wang Y., Zhang B., Yin S., Zhu H., Xing Z. (2016). Evaluation of ecosystem service value of riparian zone using land use data from 1986 to 2012. Ecol. Indic..

[25] Yi H., Güneralp B., Filippi A.M., Kreuter U.P., Güneralp I. (2017). Impacts of Land on ecosystem services in the San Antonio River Basin, Texas, from 1984 to 2010. Ecol. Econ..

[26] Ruckelshaus M., McKenzie E., Tallis H., Guerry A., Daily G., Kareiva P., Polasky S., Ricketts T., Bhagabati N., Wood S.A. (2015). Notes from the field: Lessons learned from using ecosystem service approaches to inform real-world decisions. Ecol. Econ..

[27] Lautenbach S., Kugel C., Lausch A., Seppelt R. (2011). Analysis of historic changes in regional ecosystem service provisioning using land use data. Ecol. Indic..

[28] Hu S., Chen L., Li L., Zhang T., Yuan L., Cheng L., Wang J., Wen M. (2020). Simulation of land use change and ecosystem service value dynamics under ecological constraints in Anhui Province, China. Int. J. Environ. Res. Public Health.

[29] Woldeyohannes A., Cotter M., Biru W.D., Kelboro G. (2020). Assessing changes in ecosystem service values over 1985–2050 in response to land use and land cover dynamics in Abaya-Chamo Basin, Southern Ethiopia. Land.

[30] Zhao L., Fan X., Lin H., Hong T., Hong W. (2020). Impact of Urbanization on the value of ecosystem services in Nanping City, China. Pol. J. Environ. Stud..

[31] Sutton P.C., Costanza R. (2002). Global estimates of market and non-market values derived from nighttime satellite imagery, land cover, and ecosystem service valuation. Ecol. Econ..

[32] Estoque R.C., Murayama Y. (2013). Landscape pattern and ecosystem service value changes: Implications for environmental sustainability planning for the rapidly urbanizing summer capital of the Philippines. Landsc. Urban Plan..

[33] Liu S., Yang M., Mou Y., Meng Y., Zhou X., Peng C. (2020). Effect of urbanization on ecosystem service values in the Beijing-Tianjin-Hebei urban agglomeration of China from 2000 to 2014. Sustainability.

[34] Hou Y., Zhou S., Burkhard B., Müller F. (2014). Socioeconomic influences on biodiversity, ecosystem services and human well-being: A quantitative application of the DPSIR model in Jiangsu, China. Sci. Total Environ..

[35] Wang B., Gao P., Niu X., Sun J. (2017). Policy-driven China’s grain to green program: Implications for ecosystem services. Ecosyst. Serv..

[36] Yin F., Mao F., Fu B., Liu G. (2006). Farmland ecosystem service and its formation mechanism. Chin. J. Appl. Ecol..

[37] Ling H., Yan J., Xu H., Guo B., Zhang Q. (2019). Estimates of shifts in ecosystem service values due to changes in key factors in the Manas River basin, northwest China. Sci. Total Environ..

[38] Fu Q., Li B., Hou Y., Bi X., Zhang X. (2017). Effects of land use and climate change on ecosystem services in Central Asia’s arid regions: A case study in Altay Prefecture, China. Sci. Total Environ..

[39] De Lima G.T.N.P., Hackbart V.C.D.S., Bertolo L.S., Dos Santos R.F. (2016). Identifying driving forces of landscape changes: Historical relationships and the availability of ecosystem services in the Atlantic Forest. Ecosyst. Serv..

[40] Zhao Z.G., Yu D., Han C.Y., Wang K.R. (2017). Ecosystem services value prediction and driving forces in the Poyang Lake eco-economic zone. Acta Ecol. Sin..

[41] Zhi Y., Jin Z., Lu L., Yang T., Zhou D., Pei Z., Wu D., Fu D., Zhang D., Li X. (2021). Improving atmospheric corrosion prediction through key environmental factor identification by random forest-based model. Corros. Sci..

[42] Xiao L., Zhou Y., Huang H., Liu Y.-J., Li K., Li M.-Y., Tian Y., Wu F. (2020). Application of geostatistical analysis and random forest for source analysis and human health risk assessment of potentially toxic elements (PTEs) in Arable Land soil. Int. J. Environ. Res. Public Health.

[43] Song C.S. (1994). Scientific Survey of the Funiu Mountain Nature Reserve.

[44] Xie G., Zhen L., Lu C., Xiao Y., Chen C. (2008). Expert knowledge based valuation method of ecosystem services in China. J. Nat. Resour..

[45] Xing L., Zhu Y., Wang J. (2021). Spatial spillover effects of urbanization on ecosystem services value in Chinese cities. Ecol. Indic..

[46] Shi Y., Wang R., Huang J., Yang W. (2012). An analysis of the spatial and temporal changes in Chinese terrestrial ecosystem service functions. Chin. Sci. Bull..

[47] Wang H., Qin F., Zhu J., Zhang C.C. (2017). The effects of land use structure and landscape pattern change on ecosystem service values. Acta Ecol. Sin..

[48] Verburg P.H., Erb K.-H., Mertz O., Espindola G. (2013). Land system science: Between global challenges and local realities. Curr. Opin. Environ. Sustain..

[49] Laliberté E., Wells J.A., De Clerck F., Metcalfe D.J., Catterall C.P., Queiroz C., Aubin I., Bonser S.P., Ding Y., Fraterrigo J.M. (2010). Land-use intensification reduces functional redundancy and response diversity in plant communities. Ecol. Lett..

[50] Li N., Wang J., Wang H., Fu B., Chen J., He W. (2021). Impacts of land use change on ecosystem service value in Lijiang River Basin, China. Environ. Sci. Pollut. Res..

[51] Schirpke U., Tscholl S., Tasser E. (2020). Spatio-temporal changes in ecosystem service values: Effects of land-use changes from past to future (1860–2100). J. Environ. Manag..

[52] Su C.H., Wang Y.L. (2018). Evolution of ecosystem services and its driving factors in the upper reaches of the Fenhe River watershed, China. Acta. Ecol. Sin..

[53] Sun Y.J., Ren Z.Y., Hao M.Y., Duan Y.F. (2019). Spatial and temporal changes in the synergy and trade-off between ecosystem services, and its influencing factors in Yanan, Loess Plateau. Acta. Ecol. Sin..

[54] Hong Y., Ding Q., Zhou T., Kong L., Wang M., Zhang J., Yang W. (2020). Ecosystem service bundle index construction, spatiotemporal dynamic display, and driving force analysis. Ecosyst. Health Sustain..

[55] Breiman L. (2001). Random forests. Mach. Learn..

[56] Xie C., Chao L., Shi D., Ni Z. (2020). Evaluation of sustainable use of water resources based on random forest: A Case study in the Lishui River Basin, Central China. J. Coast. Res..

[57] Chen J., Yang S.T., Li H.W., Zhang B., Lv J.R. (2013). Research on Geographical environment unit division based on the method of natural breaks (Jenks). ISPRS.

[58] Tang Z., Mei Z., Liu W., Xia Y. (2020). Identification of the key factors affecting Chinese carbon intensity and their historical trends using random forest algorithm. J. Geogr. Sci..

[59] Kong F., Nakagoshi N. (2006). Spatial-temporal gradient analysis of urban green spaces in Jinan, China. Landsc. Urban Plan..

[60] Li T., Ding Y. (2017). Spatial disparity dynamics of ecosystem service values and GDP in Shaanxi Province, China in the last 30 years. PLoS ONE.

[61] Peng W.F., Zhang D.M., Luo Y.M., Tao S., Xu X.L. (2019). Influence of natural factors on vegetation NDVI using geographical detection in Sichuan Province. Acta Geogr. Sin..

[62] Zhuo G., Chen S.R., Zhou B. (2018). Spatio-temporal variation of vegetation coverage over the Tibetan Plateau and its responses to climatic factors. Acta. Ecol. Sin..

[63] Yoshida A., Chanhda H., Ye Y.-M., Liang Y.-R. (2010). Ecosystem service values and land use change in the opium poppy cultivation region in Northern Part of Lao PDR. Acta Ecol. Sin..

[64] Li J., Wang W., Hu G., Wei Z. (2010). Changes in ecosystem service values in Zoige Plateau, China. Agric. Ecosyst. Environ..

[65] McDonnell M.J., Pickett S.T., Groffman P., Bohlen P., Pouyat R.V., Zipperer W.C., Parmelee R.W., Carreiro M.M., Medley K. (1997). Ecosystem processes along an urban-to-rural gradient. Urban Ecol..

[66] Zhang Z.H., Chen N., Zhu B., Tao H.T., Cheng H.R. (2021). Source analysis of ambient PM2.5 in Wuhan City based on random forest model. Environ. Sci..

[67] Huang H., Zhou Y., Liu Y.J., Xiao L., Li K., Duan J.S., Wei H.L. (2020). Source analysis of heavy metals in farmland based on environmental variables and random forest approach: District of Xiangzhou District in Xiangyang City. Acta Sci. Circumst..

[68] Xu J., Liu F., Wu H.Y., Song X.D., Zhao Y.G., Zhang G.L. (2021). Predicting of key environmental factors from soil properties based on artificial neural network and random forest learning model. Chin. J. Soil. Sci..

[69] Chen D.-L., Lu X.-H., Kuang B. (2019). Measurement of cultivated land utilization efficiency: Construction and application of random forest. J. Nat. Resour..

[70] Luo Q., Zhou J., Li Z., Yu B. (2020). Spatial differences of ecosystem services and their driving factors: A comparation analysis among three urban agglomerations in China’s Yangtze River economic belt. Sci. Total. Environ..

[71] Liu Y., Li J., Zhang H. (2012). An ecosystem service valuation of land use change in Taiyuan City, China. Ecol. Model..

